# Liver-directed chemotherapy of cetuximab and bevacizumab in combination with oxaliplatin is more effective to inhibit tumor growth of CC531 colorectal rat liver metastases than systemic chemotherapy

**DOI:** 10.1007/s10585-012-9550-9

**Published:** 2012-11-27

**Authors:** Jens Sperling, David Brandhorst, Thilo Schäfer, Christian Ziemann, Anna Benz-Weißer, Claudia Scheuer, Otto Kollmar, Martin K. Schilling, Michael D. Menger

**Affiliations:** 1 Department of General, Visceral, Vascular and Pediatric Surgery, Saarland University Hospital, Homburg/Saar, Germany; 2 Institute for Clinical and Experimental Surgery, University of Saarland, Homburg/Saar, Germany; 3 Present Address: Department of General and Visceral Surgery, University Medical Center, Georg August University Göttingen, Robert-Koch-Strasse 40, 37075 Göttingen, Germany

**Keywords:** Colorectal liver metastases, Hepatic arterial infusion, Systemic chemotherapy, Bevacizumab, Cetuximab, Oxaliplatin

## Abstract

Colorectal carcinoma is, through to its high rate of liver metastasis (mCRC), the second most cause of cancer death worldwide. Tumor resection represents the only potential cure. In cases of unresectable disease systemic chemotherapy (sCHT) remains the therapy of choice. Modern sCHT regimens including biological agents can induce tumor response that leads to curative surgery of initially unresectable mCRC. However, liver-directed therapy via hepatic arterial infusion (HAI) may produce higher response rates than sCHT. Herein we studied whether a HAI of cetuximab (CE) plus bevacizumab (BE) with or without oxaliplatin (OX) can inhibit tumor growth in a rat model. WAG/Rij rats underwent subcapsular hepatic tumor implantation. After 10 days animals received either HAI or sCHT of CE plus BE, OX or all three drugs. Saline-treated animals served as controls. Tumor growth was estimated at day 10 and 13. On day 13 liver and tumor tissue was studied histologically and immunohistochemically. In controls the tumors grew about 50 %. OX alone was not capable of inhibiting tumor growth. In contrast, CE plus BE given as HAI significantly reduced tumor growth compared to sCHT (*p* < 0.05). HAI of CE plus BE combined with OX yielded an even more pronounced inhibition of tumor growth. Immunohistochemistry revealed a decreased tumor cell proliferation and tumor vascularization. The present study demonstrates that HAI of CE plus BE is effective to inhibit tumor growth. This effect is even more pronounced in combination with OX. Systemic application of these agents cannot achieve comparable effects.

## Introduction

Colorectal carcinoma has a rising incidence and is the second most cause of cancer related death worldwide [1, 2]. About 50 % of patients develop metastatic disease, whereby the liver remains the most common site of metastasis. These patients have a poor prognosis demonstrated by a 5-year survival rate in the range of 5–8 %. The only potential cure is complete resection of the hepatic tumor burden [3]. Unfortunately, a great number of patients suffer from unresectable disease. In these patients the main goal is to induce tumor response by systemic chemotherapy which can lead to curative surgery of initially unresectable metastases.

During the last years, the development of new cytotoxic drugs and regimens as well as biological targeted agents like cetuximab (CE) or bevacizumab (BE) has improved the survival outcome of patients with unresectable disease [3]. Cetuximab is a monoclonal antibody directed against the ligand binding domain of the epidermal growth factor receptor (EGF-R). Bevacizumab is a monoclonal antibody directed against the vascular endothelial growth factor (VEGF). The use of these antibodies in modern sCHT regimens has improved the 2-year survival rate, however, it still does not exceed 40 %. Of interest, the analysis of the 5-year survival rate could not show a benefit of the survival outcome [3, 4].

It is well known that liver-directed chemotherapy, which is achieved by hepatic arterial infusion, yields reproducibly higher response rates compared to sCHT both in preclinical and clinical settings [2, 5]. However, the overall value of HAI remains unclear [6]. Therefore, it has been suggested to evaluate the effect of a HAI especially with the delivery of novel cancer agents or drug combinations in further studies [7]. Accordingly, we herein studied whether a HAI of cetuximab plus bevacizumab (CE + BE) alone or in combination with oxaliplatin (OX) is more effective than sCHT to inhibit tumor growth in a mCRC model of the rat liver.

## Materials and methods

### Animals

For the experiments we used 48 male WAG/Rij rats with a body weight of 248.3 ± 5.5 g (mean ± standard error of the mean (SEM)). Animals were kept in a temperature- and humidity-controlled 12 h light/dark cycle environment with free access to water and standard laboratory chow (Altromin, Lage, Germany).

### Experimental protocol

All experiments were approved by the local governmental ethic committee. Animals were randomized to eight groups (*n* = 6 each), whereby four groups of animals underwent HAI and four groups underwent sCHT. 10 days after tumor cell implantation relaparotomy was performed and animals received either (CE + BE), (OX) or all three drugs (CE + BE + OX) via HAI or sCHT. Sham animals received a comparable amount of 0.9 % saline solution (Braun, Melsungen, Germany) and served as controls (HAI sham, sCHT sham). Prior to the treatment the tumor volume was estimated by three-dimensional ultrasound imaging. Three days later animals underwent relaparotomy and reexamination of the tumor volume by three-dimensional ultrasound imaging. Finally, animals were sacrificed and tissue was sampled for histological and immunohistochemical analysis. Body weight was measured for determination of weight reduction on the day of tumor implantation as well as at the end of the experiments.

### Tumor cell implantation

Under ether anesthesia a median laparotomy was performed. Induction of colorectal liver metastases was achieved by injection of 5 × 10^5^ cells of the syngeneic CC531 rat colon carcinoma cell line (CLS, Heidelberg, Germany) under the capsule of the lower surface of the left liver lobe using a 27G needle (Omnican F, B. Braun, Melsungen, Germany). Laparotomy was closed with a one-layer running 4-0 PDS suture (Ethicon/Johnson & Johnson Medical GmbH, Norderstedt, Germany).

### Drugs

Cetuximab was administered in a dose of 114 mg/m^2^, BE in a dose of 5 mg/kg body weight and OX in a dose of 77 mg/m^2^. For the calculation of the body surface area we used “Meeh’s formula”, which reads as follows: A = K × W^2/3^. Hereby, A represents the body surface area, K an animal specific constant, which was set to 9.83 and W the individual body weight [8].

### Drug application via HAI and sCHT

On day 10 after tumor cell implantation, animals were relaparotomized under ether anaesthesia. For the HAI the gastroduodenal artery was cannulated (ID 0.28 mm, Portex, Hythe, UK), whereby the tip of the catheter was positioned at the entrance to the common hepatic artery. During the HAI the artery showed orthograde blood flow with no signs of occlusion. After HAI the catheter was removed and the gastroduodenal artery was ligated. For sCHT the subhepatic vena cava was punctured with a 23G needle (Troge Medical GmbH, Hamburg, Germany) according to previously published standards [5].

### Three dimensional ultrasound imaging

**Fig. 1:**
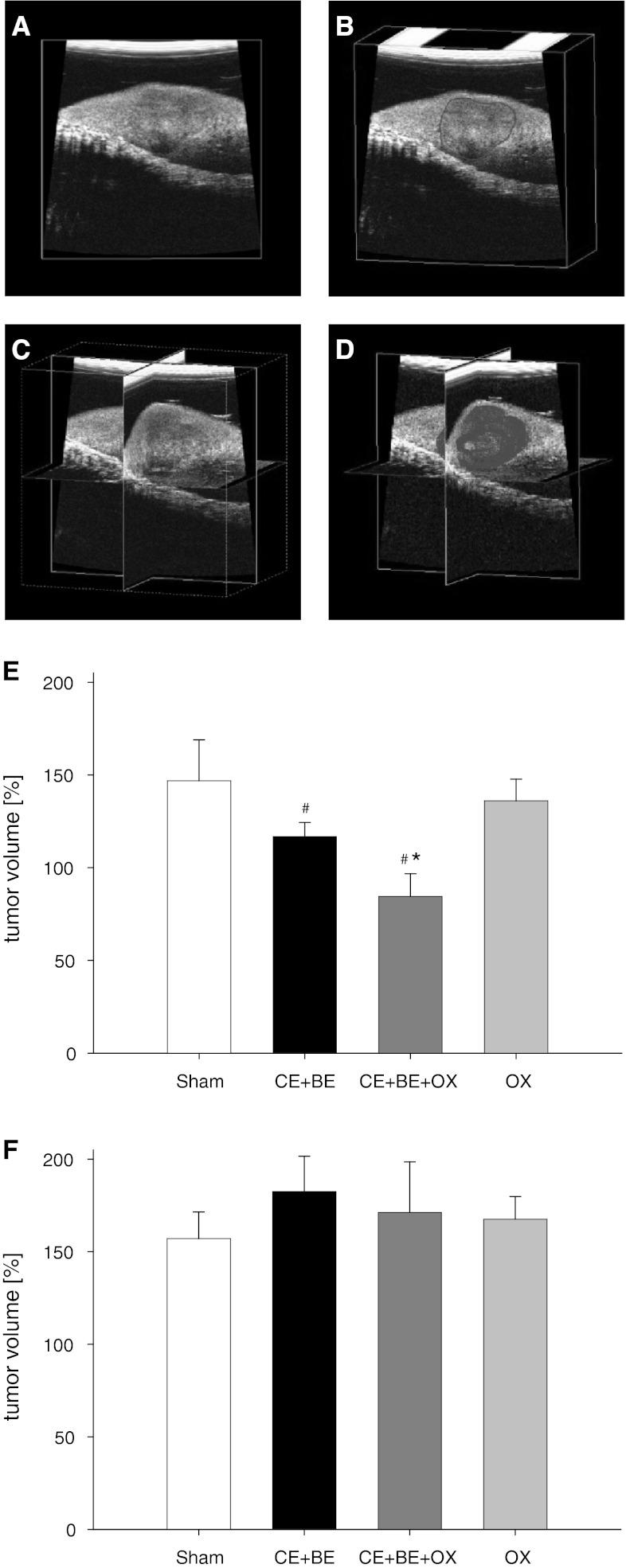
*Panels* **a**–**d** display the different phases of the three dimensional ultrasound imaging of the tumor (**a** Ultrasound image of the tumor bearing left liver lobe. **b** Off-line outlining of the tumor (*red line*), **c** Three dimensional cube, **d** Three dimensional cube with polygonal image of the tumor). The Tumor volume was measured on day 13 in percent of the volume measured on day 10 after HAI (**e**) and sCHT (**f**) of saline (sham), cetuximab plus bevacizumab (CE + BE) and oxaliplatin (OX) or the combination of all three drugs (CE + BE + OX). Data are given as mean ± SEM; *p < 0.05 versus sham; ^#^ *p* < 0.05 versus corresponding sCHT

Using the 40 MHz ultrasound probe of the Vevo 770 high-resolution imaging system (VisualSonics, Inc., Toronto, Ontario, Canada) the tumor volume was measured on day 10 and 13. The 40 MHz ultrasound probe was attached to a stepping motor that moved the probe over the surface of the left liver lobe. Thus, parallel two dimensional images were acquired in intervals of 50 μm. The tumor dimension was outlined off-line on every 200 μm of the two dimensional images. With this data, the integrated software of the Vevo 770 high-resolution imaging system calculated a polygonal three dimensional image and the tumor volume (Fig. 1a–d).

### Sampling and assays

Venous blood samples were taken at day 10 and 13 via puncture of the subhepatic vena cava. Aspartate aminotransferase (ASAT), alanine aminotransferase (ALAT) and glutamate dehydrogenase (GLDH) serum activities were analyzed using routine spectrophotometric techniques and served as indicators of hepatocellular injury. White blood cell count was determined with the use of a cell counter.

### Histology

Tissue samples of the liver were fixed in 4 % phosphate-buffered formalin and then embedded in paraffin. Sections of 5 μm stained with hematoxylin-eosin (HE) were used to perform analyses of hepatocellular vacuolization, venular endothelial detachment and venular fibrin clotting. Hepatocellular vacuolization was estimated using a semiquantitative score (0 = none; 1 = mild; 2 = moderate and 3 = severe). Venular endothelial detachment was analyzed by counting the number of venules with detachment of endothelial lining cells and documented in percent of all venules analyzed. Fibrin clotting was determined by counting the number of venules with fibrin clots and documented in percent of all venules analyzed.

### Immunohistochemical analysis

Cleaved caspase-3 (cysteine-aspartic proteases) served as an indicator of apoptotic cell death. Sections of 5 μm of tumor-bearing specimens were incubated overnight at room temperature with a rabbit polyclonal anti-cleaved caspase-3 antibody (1:50, Cell Signaling Technology, Frankfurt, Germany). As secondary antibody a peroxidase-conjugated goat-anti-rabbit-IgG antibody (1:100, Dianova, Hamburg, Germany) was used. 3.3′ diaminobenzidine served as chromogen. Counterstaining was performed with hemalaun. Per specimen 25 high-power-fields (HPF) were analyzed and positively stained cells were counted and given as number per HPF.

For the estimation of cell proliferation PCNA (proliferating cell nuclear antigen) was analyzed. Therefore, paraffin-embedded specimens sectioned in 5 μm intervals were incubated for 18 h at 4 °C with a mouse monoclonal anti-PCNA antibody (1:50; Dako, Hamburg, Germany). For development of PCNA, a peroxidase-conjugated goat anti-mouse IgG antibody (1:100; Dianova) was incubated for 30 min. 3.3′ diaminobenzidine served as chromogen. Hemalaun was used for counterstaining. PCNA-positive cells were analyzed using the following score: 0 ≤ 1 %, 1 = 1–10 %, 2 = 10–30 %, 3 = 30–50 %, 4 ≥ 50 % of PCNA-positive cells.

PECAM-1 (platelet-endothelial cell adhesion molecule-1) was used as an indicator for vascularization. The immunohistochemical detection of PECAM-1 expression was achieved using a primary mouse-anti-rat antibody (1:500; clone TLD-3 A12, Serotec, Düsseldorf, Germany) and a secondary peroxidase-conjugated goat-anti-mouse antibody (Dianova). PECAM-1-positive blood vessels were counted in 25 HPF per section and are given as number per HPF.

### Statistical analysis

After analysis of normal distribution and homogeneity of variance of the data, differences between the four HAI and the four sCHT groups were calculated separately by one-way analysis of variance (ANOVA) followed by an adequate post hoc-test (Student–Newman–Keuls test), including the correction of the alpha-error according to Bonferroni probabilities to compensate for multiple comparisons. The pairwise comparison between the HAI groups and the respective sCHT groups was performed by Student’s *t* test including a correction of the alpha-error according to Bonferroni–Holm. Statistical significance was set at *p* < 0.05 and changed according to the Bonferroni–Holm procedure. All values are expressed as mean ± Standard error of the mean SEM.

## Results

### Metastatic tumor establishment, general health conditions and body weight

10 days after tumor implantation all animals showed a solitary tumor of 5–10 mm in diameter in the left liver lobe. There were no signs of extrahepatic disease. Animals were not affected by the tumor as indicated by normal feeding and cleaning habits.

Analysis of the body weight of the sham groups demonstrated a slight but significant difference between HAI- and sCHT-treated animals between day 0 and 13, whereas between the other groups no significant differences were found (Table 1). Of interest, almost all groups receiving drug application showed a significant difference compared to the corresponding sham controls, irrespective of whether a HAI or sCHT was performed (Table 1).

**Table 1:** Change of body weight (%) from day 0 to 13

Group	HAI	sCHT
Sham	−2.1 ± 0.4^#^	+8.3 ± 1.2
CE + BE	−1.4 ± 1.2	−3.4 ± 1.0*
CE + BE + OX	−6.8 ± 0.8*	−7.8 ± 1.9*
OX	−6.8 ± 1.8*	−0.9 ± 3.7*

Change of body weight from day 0 to 13 (given in percent). Animals were treated either with hepatic arterial infusion (HAI) or systemic application (sCHT) of saline (sham), cetuximab plus bevacizumab (CE + BE), the combination of cetuximab, bevacizumab and oxaliplatin (CE + BE + OX) or oxaliplatin alone (OX). Data are given as mean ± SEM

* *p* < 0.05 versus sham; ^#^ *p* < 0.05 versus corresponding sCHT

### Metastatic tumor growth

Ultrasound analysis in both HAI- and sCHT-sham animals revealed an almost 50 % increase of tumor volume from day 10 to 13 (Fig. 1). HAI or sCHT of OX alone induced only a slight but not significant decrease of tumor growth compared to sham controls. Of interest, HAI of the monoclonal antibodies (HAI CE + BE) induced a greater inhibition of tumor growth during the three-day period. Moreover, the combination of the monoclonal antibodies with OX given via HAI (HAI CE + BE + OX) not only inhibited tumor growth, but significantly reduced the tumor size (Fig. 1). In contrast, systemic application (sCHT) of CE + BE and CE + BE + OX was not capable of affecting the tumor growth (Fig. 1).

### Tumor cell proliferation

**Fig. 2:**
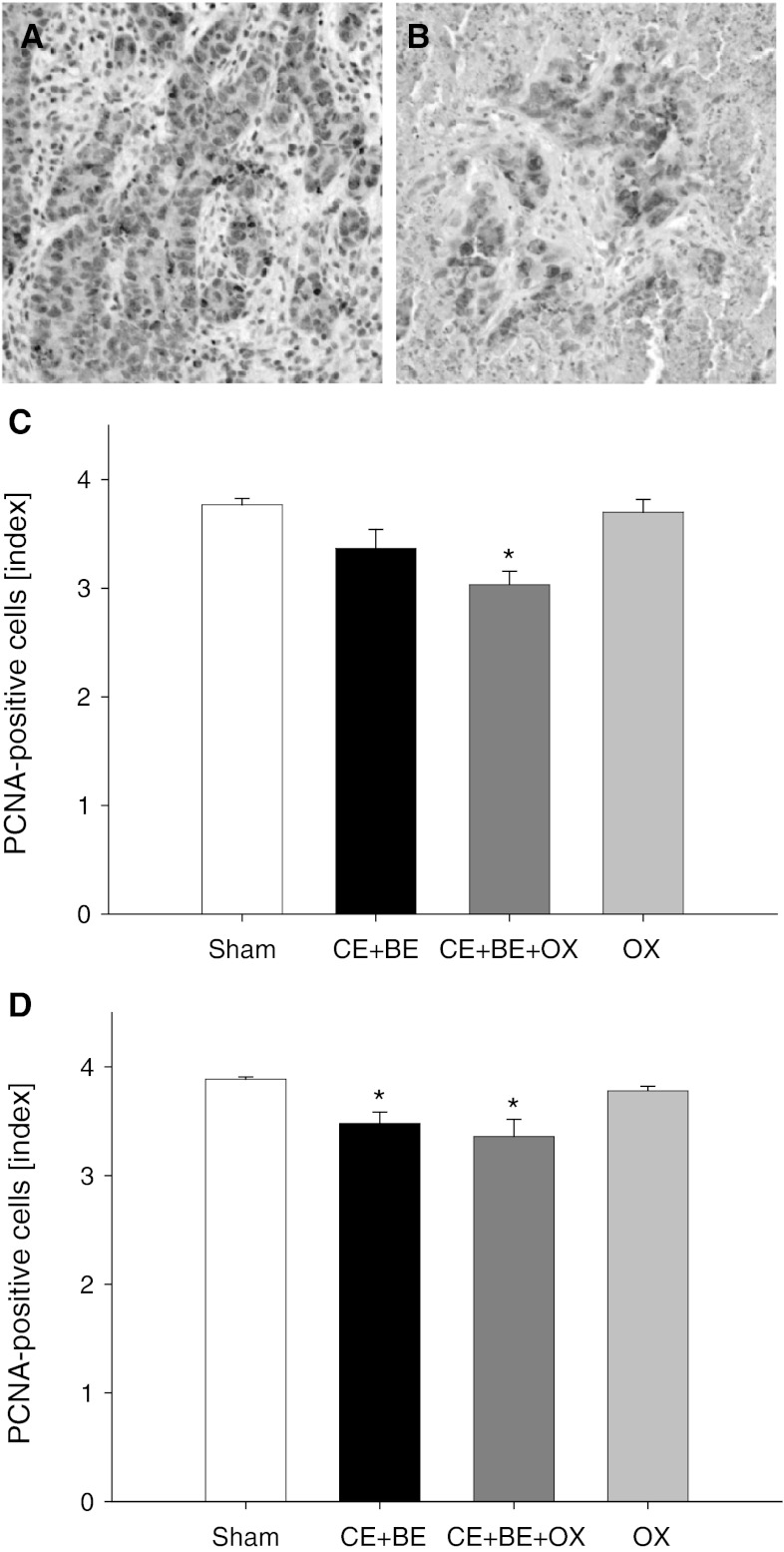
Immunohistochemical sections of proliferating nuclear cell antigen (PCNA) expression in the tumor tissue (**a** and **b**). The PCNA-positive cells are stained *brown* (**a** HAI sham group, **b** HAI CE + BE + OX). *Panels* **c** and **d** show the data of the immunohistochemical analysis of PCNA (given as index: 0≤ 1 %, 1 = 1–10 %, 2 = 10–30 %, 3 = 30–50 %, 4 ≥ 50 % of PCNA-positive cells) in the tumor of animals undergoing HAI (**c**) or sCHT (**d**) of cetuximab plus bevacizumab (CE + BE), oxaliplatin (OX) or the combination of all three drugs (CE + BE + OX). Animals undergoing HAI or systemic application with saline served as controls (sham). Data are given as mean ± SEM; *p < 0.05 versus sham. ×200 magnification

Immunohistochemical analysis of tumor cell proliferation revealed up to 50 % PCNA-positive cells in sham controls of HAI- and sCHT-treated animals (Fig. 2). Application of OX alone showed almost similar results (Fig. 2). In contrast, CE + BE but in particular the combination of CE + BE with OX (CE + BE + OX) significantly lowered the number of proliferating tumor cells compared to sham controls. This effect was most pronounced in animals treated with HAI of CE + BE + OX (Fig. 2).

### Apoptotic tumor cell death

**Fig. 3:**
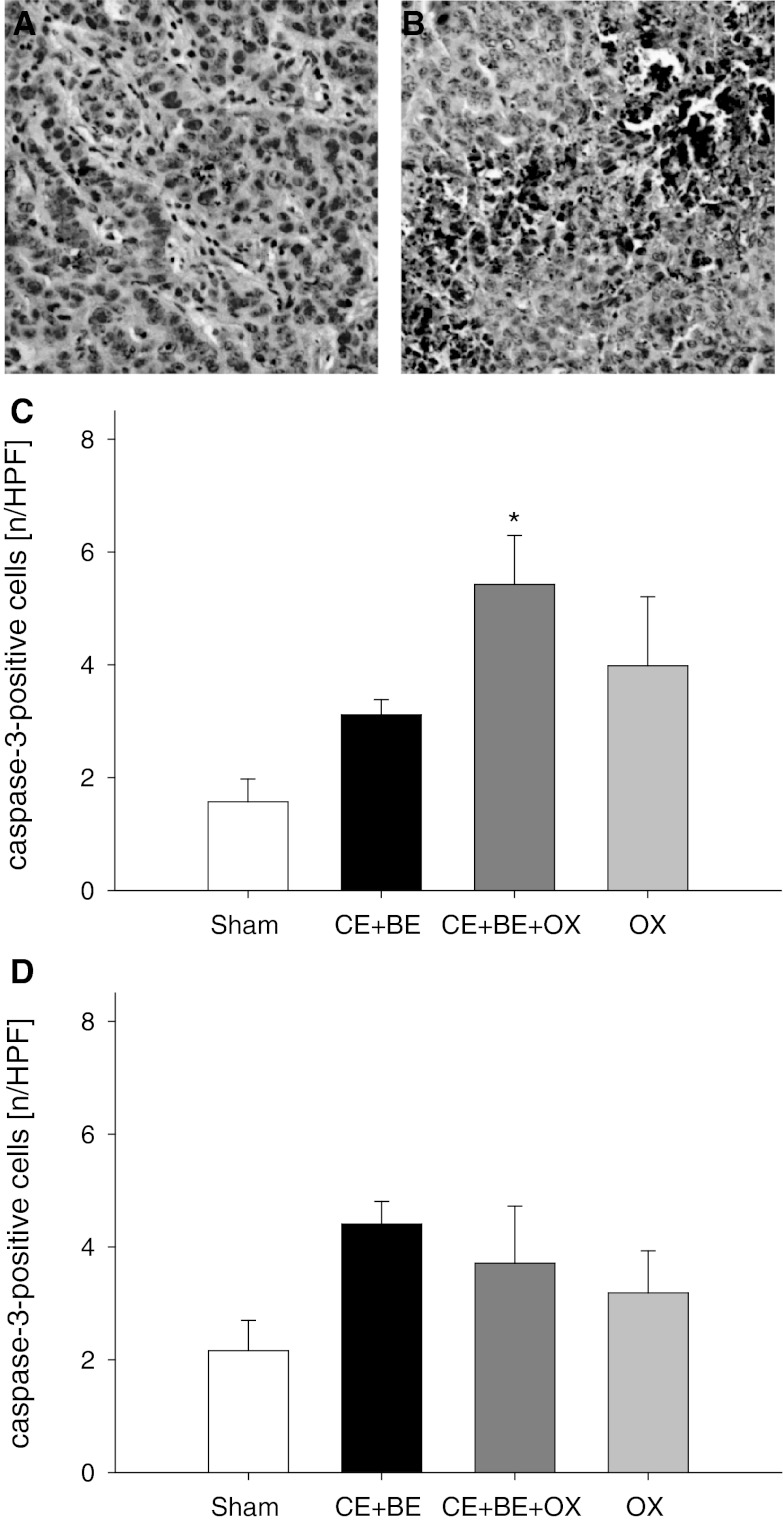
Immunohistochemical sections of cleaved caspase-3 as an indicator of apoptotic cell death (**a** and **b**). Apoptotic cells are stained *brown* (**a** sCHT CE + BE + OX, **b** HAI CE + BE + OX). *Panels* **c** and **d** display the quantitative analysis of cleaved caspase-3-positive cells in the tumor (given as number per HPF) of animals undergoing HAI (**c**) or sCHT (**d**) of cetuximab plus bevacizumab (CE + BE), oxaliplatin (OX) or the combination of all three drugs (CE + BE + OX). Animals undergoing HAI or systemic application of saline served as controls (sham). Data are given as mean ± SEM; *p < 0.05 versus sham. ×200 magnification

Analysis of cleaved caspase-3-positive cells showed a slight but not significant increase of apoptotic cell death in the tumor tissue after CE + BE and also after OX alone compared to sham controls (Fig. 3). However, the combination of all three agents (CE + BE + OX) when given via HAI significantly increased the number of apoptotic cells by more than three-fold compared to HAI sham controls. In contrast, the number of apoptotic cells after sCHT CE + BE + OX was not significantly increased compared to sCHT sham (Fig. 3).

### Tumor vascularization

**Fig. 4:**
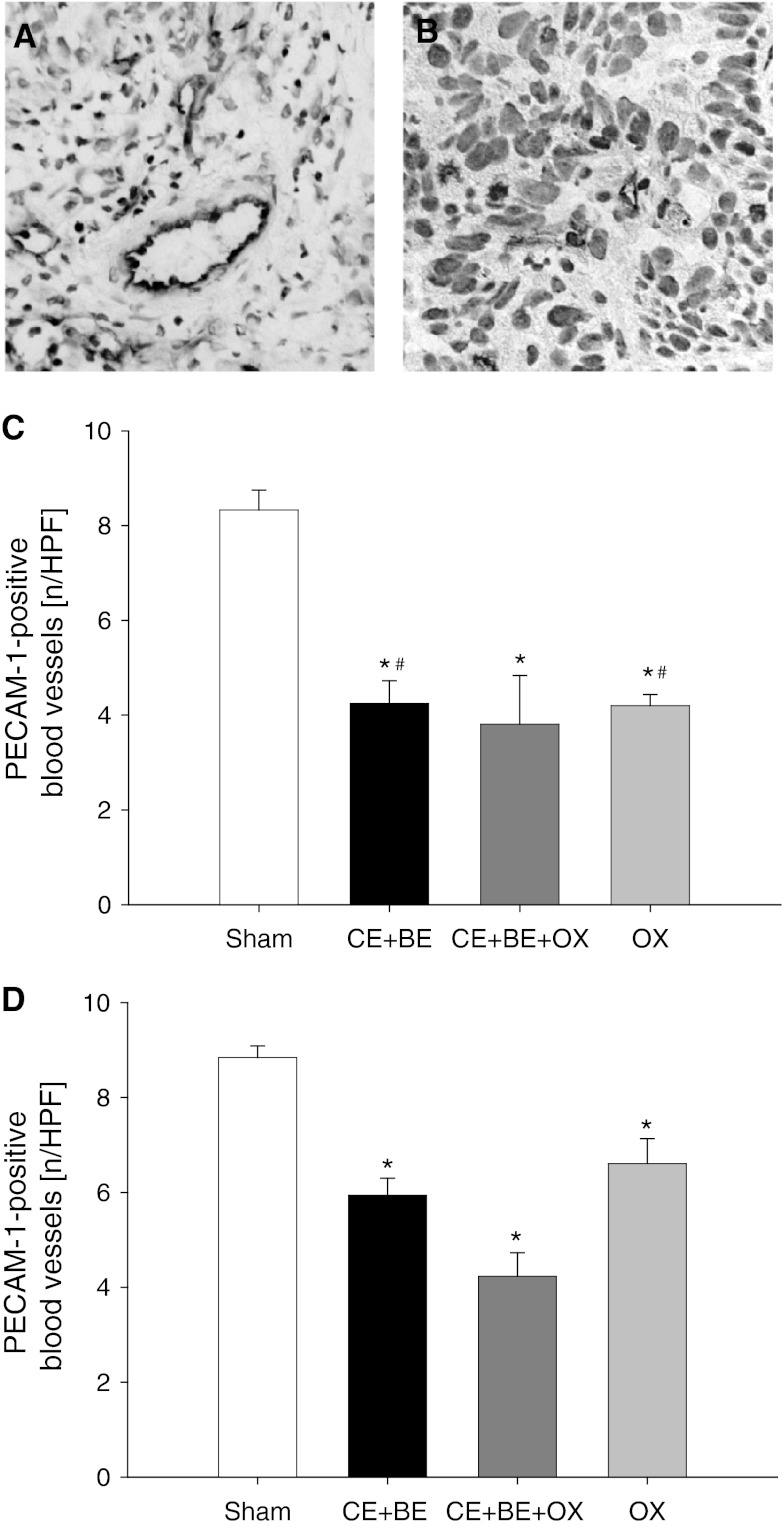
Immunohistochemical sections of PECAM-1-positive blood vessels of the tumor tissue (**a** and **b**). PECAM-1-positive blood vessels are stained *brown* (**a** HAI sham, **b** HAI CE + BE + OX). *Panels* **c** and **d** show the quantitative analysis of PECAM-1-positive blood vessels in the tumor (given as number per HPF) of animals undergoing HAI (**c**) or sCHT (**d**) of cetuximab plus bevacizumab (CE + BE), oxaliplatin (OX) or the combination of all three drugs (CE + BE + OX). Animals undergoing HAI or systemic application of saline served as controls (sham). Data are given as mean ± SEM; *p < 0.05 versus sham; ^#^ *p* < 0.05 versus corresponding sCHT. ×400 magnification

HAI of CE + BE, CE + BE + OX and OX alone significantly reduced the number of PECAM-1-positive blood vessels by ~50 % compared to sham controls. The reduction of PECAM-1-positive blood vessels was found most pronounced in animals receiving CE + BE + OX (Fig. 4). Of interest, although sCHT of CE + BE, CE + BE + OX and OX alone were capable of decreasing the number of blood vessels, this effect was less pronounced compared to that observed in HAI-treated animals (Fig. 4).

### Histomorphological analysis

Vacuolization of the hepatocytes was overall moderate, although it was most pronounced after sCHT of OX (data not shown). Endothelial detachment or vascular fibrin clotting was overall negligible (data not shown). Thus, histological analysis could not reveal significant differences between HAI or sCHT treatment.

### Blood cell analysis

**Fig. 5:**
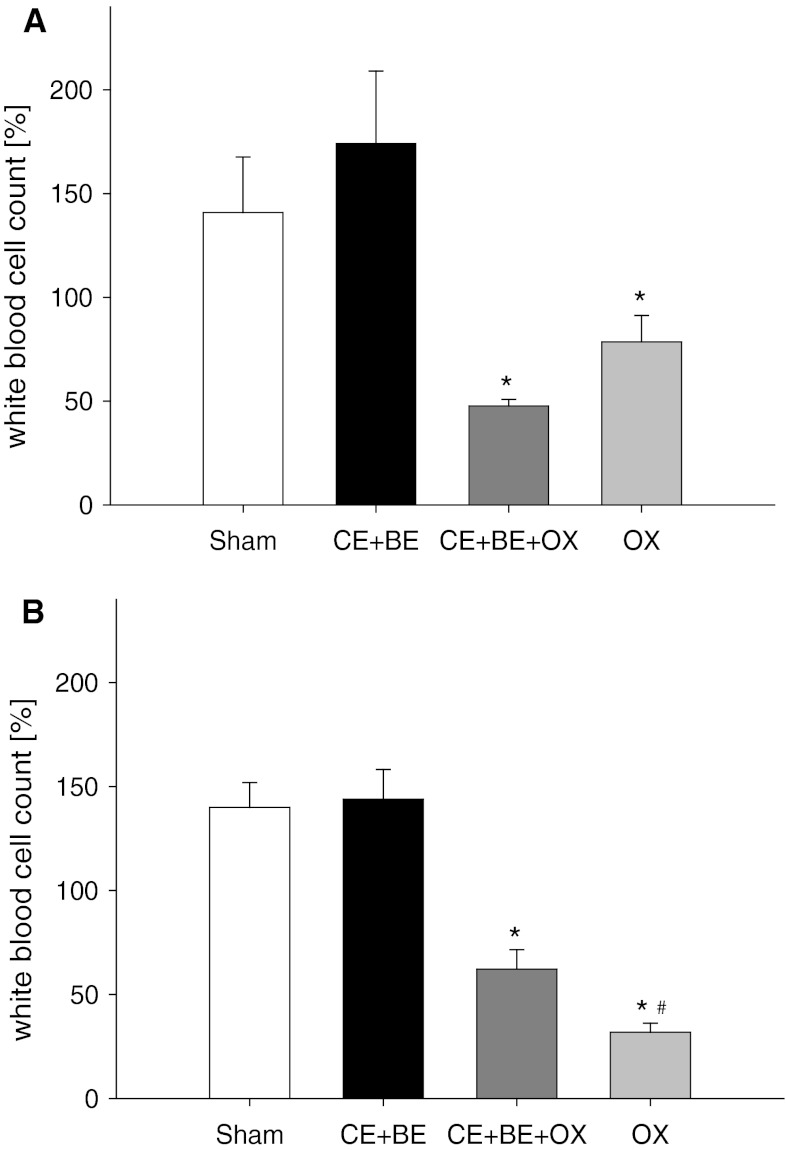
*Panels* **a** and **b** show the analysis of white blood cell count of animals undergoing HAI (**a**) or sCHT (**b**) of cetuximab plus bevacizumab (CE + BE), oxaliplatin (OX) or the combination of all three drugs (CE + BE + OX). Animals undergoing HAI or systemic application of saline served as controls (sham). Data are given as mean ± SEM; *p < 0.05 versus sham; ^#^ *p* < 0.05 versus corresponding sCHT

Analysis of white blood cell count showed a decrease of leukocytes from day 10 to 13 when OX was given alone or in combination with the monoclonal antibodies. The decrease of the number of white blood cells was found most pronounced after systemic application of OX (Fig. 5). Application of the antibodies only, regardless whether systemically or via HAI, did not affect white blood cell count.

### Analysis of liver enzymes

Analysis of the liver enzymes ASAT and ALAT as indicators of hepatocellular injury did not show any relevant differences between HAI or sCHT groups. Of interest, GLDH as an indicator of mitochondrial damage was slightly increased after HAI of CE + BE + OX and OX alone compared to the respective sCHT groups (data not shown).

## Discussion

The major finding of the present study is that a HAI of the monoclonal antibodies CE and BE is effective to inhibit tumor growth in a colorectal rat liver metastasis model. Moreover, the combination of CE and BE with OX given via HAI not only inhibited tumor growth, but was even capable of decreasing the tumor size. Of interest, these effects could not be observed when the drugs were applied as sCHT.

Colorectal cancer has a rising incidence worldwide and is, today, the leading cause of cancer death in Germany with an incidence of ~60,000 per year [1, 9]. The prognosis is mostly determined by its high rate of liver metastases. About 20 % of patients show synchronous hepatic metastases and 20–30 % develop metachronous disease [9]. However, due to unresectable disease or limiting comorbidities liver resection is only feasible in less than 25 % of the patients [10]. Because liver resection represents the only curative approach, therapeutic strategies are mandatory to primary downsize the tumor and make surgery feasible in as many patients as possible.

Systemic chemotherapy (sCHT) remains the therapy of choice in the treatment of unresectable mCRC. The most common algorithms comprise 5-fluorouracil plus leucovorin in combination with irinotecan or OX (FOLFIRI and FOLFOX). These are reported to enable a resection rate of 9–40 % among patients with initially unresectable disease [11, 12]. The integration of targeted agents, such as the monoclonal antibodies CE and BE, has led to a further improvement of the patients outcome [13]. Cetuximab is known to be capable of increasing the resectability rate in patients with primary unresectable disease refractory to conventional first-line therapy [14]. In several clinical studies BE has also been proven to increase the resectability rate [15–17]. Whether the combination of CE and BE can lead to a further improvement remains to be determined and is part of current research [18, 19].

However, beside the contents of the chemotherapy regimens, today, there is an ongoing discussion whether the efficacy of chemotherapy can be augmented by changing the route of delivery. Hepatic arterial infusion as a liver-directed loco-regional drug application is capable of increasing the local concentration of a specific tumoricidal agent compared to sCHT and may thus increase its anti-tumor activity [20]. Although higher response rates were reported using HAI with different drugs, this did not translate into a significant survival benefit for the patients [7]. Thus, current reviews came to the conclusion that the value of HAI needs to be newly investigated with the use of novel agents like CE, BE and OX [2, 6, 7].

In the present study the systemic application of CE and BE did not inhibit tumor growth of CC531 colorectal rat liver metastases. In contrast, the liver-directed therapy of these agents led to a significant inhibition of tumor growth. The major anti-tumor effect of CE and BE is related to the inhibition of angiogenesis. CE is a monoclonal human/murine chimeric antibody against the EGF-R, which is overexpressed in colorectal cancer [21–23]. Deregulation of EGF-R decreases tumor proliferation, angiogenesis and resistance to apoptosis [24].

BE is a recombinant humanized monoclonal antibody, targeting the VEGF by binding its soluble form, which prevents the ligand from binding to the VEGF receptor (VEGF-R) [25]. The VEGF/VEGF-R system plays a major role in angiogenesis and, especially, metastatic colorectal cancer has been shown to involve VEGF expression [26]. The tumoricidal effects of BE were shown in several preclinical studies, describing effective inhibition of angiogenesis, induction of apoptosis and reduction of proliferation [27–35]. As single agents, as well as in combination with OX, both CE and BE already showed their anti-tumor activity given as HAI in a rodent model of liver metastases [5, unpublished data].

However, due to the different modes of action it may be speculated that CE and BE together provide an additive anti-tumor effect. The evaluation of the short-term effects in the present study demonstrates that a single HAI of the drug combination CE plus BE decreases tumor cell proliferation and increases the rate of apoptotic cell death in the tumor tissue. Moreover, the analysis of PECAM-1 expression revealed a reduction of the number of tumor blood vessels by ~50 % after HAI of CE plus BE. In accordance, a considerable inhibition of tumor growth was detected. These effects were even more pronounced when CE + BE were combined with OX.

Oxaliplatin, a well known cytostatic drug, is a diaminocyclohexane platinum, which provides a large spectrum of anti-cancer activity [36–43]. In a preclinical study Dzodic et al. [36] found a significant pharmacokinetic advantage when OX was given as HAI compared to systemic i.v. administration. Clinical studies have underlined this finding, showing promising results without major toxicity in the treatment of colorectal liver metastases with OX-based HAI therapy [44–46]. Accordingly, in the present study we detected an increased anti-tumor effect when OX was added to CE + BE. This is illustrated by the fact that the anti-proliferative, pro-apoptotic and anti-angiogenic effects were found most pronounced after HAI of CE + BE + OX. As a result, this therapeutic regime not only inhibited tumor growth but additionally induced tumor destruction.

Accordingly, we assume that the mechanisms of action inhibiting tumor growth after HAI of CE + BE + OX are multifactorial, including the anti-angiogenic properties of the drugs, but also direct anti-proliferative and pro-apoptotic actions.

This view is in line with in vitro data from others. Balin-Gauthier and coworkers [47] showed that CE potentiates the OX-mediated cytotoxic effect as a result of inhibition of nucleotide excision repair and also DNA replication initiation. Comparable results were found by Fan et al. for anti-VEGF-therapies. The authors showed that the exposure of human colorectal cancer cells to OX led to a marked induction of VEGF and concluded, that the neutralization of pro-survival responses with anti-VEGF therapy might explain some of the beneficial effects of anti-VEGF therapy when added to chemotherapy [48].

Of interest, it is known that the drug combination of CE + BE + OX is not necessarily associated with greater side effects when compared with those observed after single application of the drugs [49]. In line with this, we did not observe toxic side effects in the HAI-treated animals and sCHT controls during the 3 day follow up period in the present study.

Taken together, our short-term analysis in a rodent model of colorectal liver metastases demonstrates that HAI of CE + BE + OX is more effective to inhibit tumor growth compared to sCHT of these drugs.
